# Efficacy and safety of Yinchen Sini decoction in treating biliary atresia patients after Kasai portoenterostomy: A systematic review and meta-analysis protocol

**DOI:** 10.1097/MD.0000000000013935

**Published:** 2019-01-11

**Authors:** Guoming Chen, Chuyao Huang, Jiaxin Lu, Ruilan Huang, Jie Zhang, Ziyin Chen, Hua Xu

**Affiliations:** a Guangzhou University of Chinese Medicine, Guangzhou, China; b Department of paediatrics, First Affiliated Hospital of Guangzhou University of Chinese Medicine, Guangzhou, China.

**Keywords:** biliary atresia, Kasai portoenterostomy, protocol, systematic review, Yinchen Sini decoction

## Abstract

**Background:**

Biliary atresia (BA) is a neonatal obstructive biliary tract disease in which the intrahepatic and extrahepatic bile ducts are obstructed and can lead to congenital biliary atresia of cholestatic cirrhosis and eventually liver failure. It has been confirmed that the Kasai portoenterostomy is an effective treatment for BA. But most patients still face complications, such as cholangitis and liver fibrosis. Yinchen Sini decoction (YCSND), a traditional herbal formula, is used as a treatment for BA after Kasai portoenterostomy. And it is supported that YCSND can improve jaundice and liver fibrosis through multiple targets and pathways. Based on the published literature, this study aims to evaluate the current situation in the treatment of BA in children with YCSND.

**Methods:**

The following databases will be searched until October 2018: PubMed, The Cochrane Library, Embase, Web of Science, China National Knowledge Infrastructure (CNKI), Chinese biomedical literature database (CBM), Wan Fang Database, Chinese Scientific Journals Database (VIP) and other sources such as Hand searching, Conference proceeding, International Clinical Trials Registry Platform and Chinese Clinical Trials Registry. All randomized controlled trials (RCTs) of YCSND or related formula as a treatment for postoperative patients of Kasai portoenterostomy for BA will be collected. Data extraction and risk of bias assessments will be carried out by 2 verifiers independently. The risk of bias will be evaluated through the Cochrane risk of bias tool. Review Manager software (RevMan V.5.3.0) and STATA 15 will be used for statistical analyses.

**Results:**

This study will provide a high-quality synthesis of current evidence of YCSND in treating children undergoing Kasai portoenterostomy for BA from several aspects.

**Conclusion:**

The conclusion of the meta-analysis will offer evidence for deciding whether YCSND is an effective measure for children undergoing Kasai portoenterostomy for BA.

**Ethics and dissemination:**

Not only will this systematic review be published in a peer-reviewed journal, but it will also be propagated electronically and in print. The review will bring patients benefit and provide practitioners reference in the fields of conventional medicine.

**PROSPERO registration number:**

PROSPERO CRD 42018111321.

## 1 Introduction

Biliary atresia (BA) is the most common cause of obstructive jaundice in infancy.^[1]^ Its prevalence in Europe and North America ranged from 0.5 to 0.8 per 10,000 live births, while frequency about 1 in 5000 in Taiwan.^[2,3]^ With unclear etiology and poorly defined pathogenesis, it was supported by clinical evidences that BA involves in inflammatory, viral infection, environmental, and abnormal duct development.^[4]^ Obstructed extrahepatic bile ducts and typically hyperplastic intrahepatic bile ducts cause jaundice, extensive inflammation, fibrosis and etc.^[5]^ And the progressive destruction eventually induces end-stage cirrhosis which will threaten lives even lead to death by age 2 years.^[6]^ To restore bile flow through biliary reconstruction and delay the progression of primary liver transplantation,^[7]^ the Kasai portoenterostomy was first used for BA in 1959 and has become effective treatment that increases the survival rate of BA.^[8]^ However, most of the postoperative experience several complications,^[9]^ including viral infection, cholangitis or bile lake formation,^[10]^ and finally require liver transplantation. Therefore, it is important to find effective treatment for postoperative of the Kasai portoenterostomy. Clinical evidence^[7,11–13]^ and a meta-analysis^[14]^ reported that steroids (dexamethasone, prednisolone, et al) can improve cholangitis and liver fibrosis via down-regulating serum cytokines and adhesion molecules. However, it has not confirmed whether glucocorticosteroid use in infants with BA following Kasai portoenterostomy for long-term outcomes of all-cause mortality or liver transplantation.^[13]^

Chinese herbal medicine is widely used in East Asian countries for its significant effects. The Yinchen Sini decoction (YCSND), composed by Artemisiae Scopariae Herba (yin chen), Zingiberis Rhizoma (gan jiang), Aconiti Lateralis Radix Praeparata (fu zi), Glycyrrhizae Radix Et Rhizoma (gan cao), is useful for following indications, such as liver failure and fibrosis in BA via down-regulating the expression levels of Smad3, Collagen3, Matrix metalloproteinase inhibitors (such as TIMP1, TIMP2) and Serum index of liver fibrosis (hyalaronic acid, lamini, type IV collagen, procollagen III).^[15–17]^

Based on current clinical researches, the objective of this meta-analysis is to accurately evaluate the quality of randomized controlled trials (RCTs) and obtain evidence to assess whether TCM therapy can help infants with BA following Kasai portoenterostomy.

## 2 Methods

### 2.1 Ethics and dissemination

The study has no need for ethics approval because it is not a clinical study. The informed consent is also needless because the personal information is not involved in the study. The review will be published in a peer-reviewed journal and proposed at a related conference.

### 2.2 Criteria for considering studies for this review

#### 2.2.1 Types of studies

The study will include all randomized controlled trials of YCSND for the management of postoperative patients of Kasai portoenterostomy for BA. There is no restriction on language or publication status. RCTs will be considered acceptable when YCSND is used to compare with the western medicine no matter what group YCSND is applied to.

#### 2.2.2 Types of participants

This study will include patients who were undergoing the Kasai portoenterostomy for BA.

#### 2.2.3 Types of interventions

The intervention group can use any types of YCSND including the main ingredients, which can be decoction, granules, or any other types. The formula should be conformed to the traditional principle of composition, decocting method and administration. Conventional western medicine is used as the comparison intervention.

### 2.3 Types of outcome measures

#### 2.3.1 Primary outcomes

The indexes of serum liver fibrosis in the 2 groups before and after treatment: hyalaronic acid, laminin, type IV collagen, procollagen III.

#### 2.3.2 Secondary outcomes

Colour dopplar ultrasound for the liver and spleen: the diameter of hepatic main portal vein, blood flow volume of hepatic portal vein, thickness of the spleen and the diameter of splenic vein.

### 2.4 Search methods for the identification of studies

#### 2.4.1 Electronic searches

There are 4 English databases, PubMed, Embase, the Cochrane Central Register of Controlled Trials (Cochrane Library), and Web of Science, and 4 Chinese databases, China National Knowledge Infrastructure (CNKI), Chinese Biomedical Literature Database (CBM), Chinese Science and Technology Periodical database (VIP), and Wangfang. All the databases above will be searched comprehensively from their establishment to October 2018 for the RCTs related to YCSND for the postoperative patients of the Kasai portoenterostomy for BA by our researchers. The medical subject headings and keywords will be used to develop the strategies. There is a detailed strategy for PubMed in Table 1 and it will be modified to search other databases.

**Table 1:**
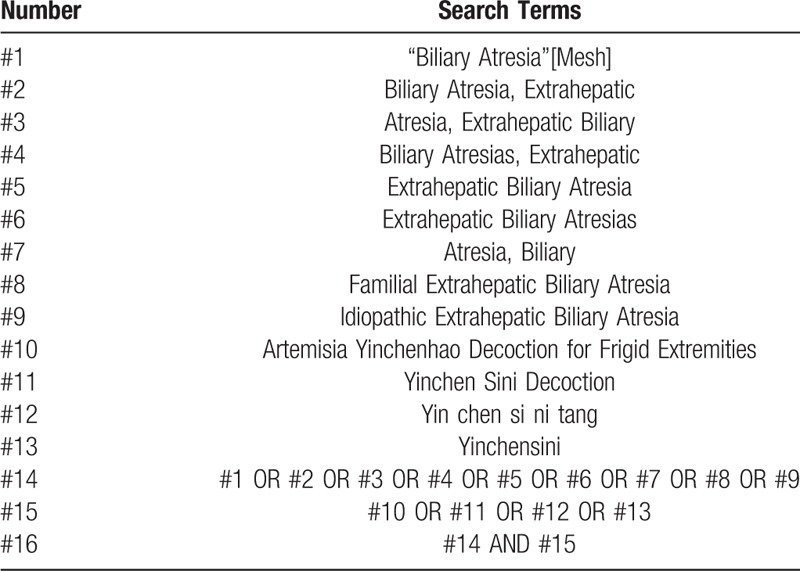
Search strategy for the PubMed database.

#### 2.4.2 Other sources

For the purpose that all the eligible literature can be searched, we will electronically or manually scan the conference proceeding, International Clinical Trials Registry Platform and Chinese Clinical Trials Registry.

### 2.5 Data collection and analysis

#### 2.5.1 Selection of studies

**Figure 1:**
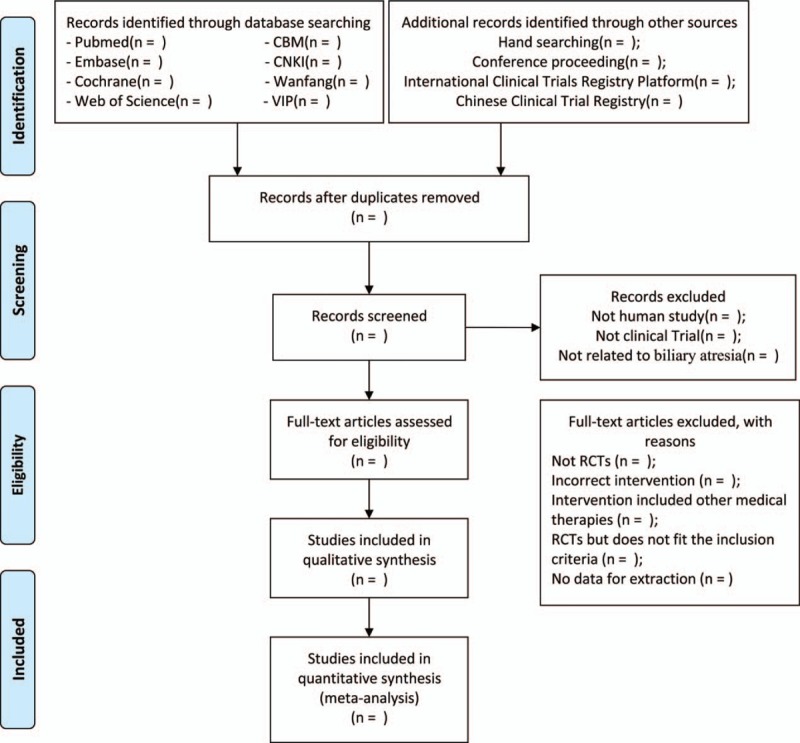
PRISMA flow diagram to describe the search process.

In order to find out all the eligible literature, 2 researchers (JL and RH) will use Endnote X9 to remove the repetitive literature and the disqualified articles by full texts after importing all papers, which are based on the inclusion and exclusion criteria (Fig. 1). All the work above will be done by the 2 researchers independently. If there is any disagreement, the researchers will resolve them by discussing with another researcher (CH).

#### 2.5.2 Data extraction and management

We will make the data extraction according to the eligible trials, first author, the date of publication, the details of the researches including the characteristics of patients, interventions and comparisons, sample size, outcomes, and adverse events, etc. The process will be done by the other 2 researchers (JL and RH) independently by using the data extraction table prepared in advance, and the third author (CH) will deal with the disagreements.

#### 2.5.3 Assessment of risk of bias in included studies

Two professional reviewers will use The Cochrane's tool recommended by Cochrane Handbook for systematic Reviews of Interventions to assess the risk of bias in the RCTs. The items are included: random sequence generation, allocation concealment, blinding method for participants and personnel, blinding of outcome assessments, incomplete data, selective reports, and other biases. The results will be regarded as low risk, unclear risk and high risk. The problem will be solved by the discussion or the arbitration with a third party when the disagreement appears.

#### 2.5.4 Unit of analysis issues

For crossover trials, we will use the data from the first period. For the trials assessing more than 1 group, the data from each control group will be combined with the main analysis. We will perform the subgroup analysis of the control groups and count every patient only once.

#### 2.5.5 Dealing with missing data

The reviewers will contact the original author for the sufficient information, if the data is unclear or lacking. If the researchers cannot contact the author, the rest of available data will be analyzed for the outcome, and we will evaluate the potential influence of the missing data on the conclusion.

#### 2.5.6 Assessment of heterogeneity

We will use *I*^*2*^ to evaluate the heterogeneity. If *I*^*2*^ is more than 50%, we regard that there is a significant heterogeneity. Subsequently, we manage a descriptive statistical analysis for data synthesis and detect the latent factors by using the subgroup analysis. If *I*^*2*^ is less than 50%, it means that heterogeneity is low and then the Chi-squared test is used to search for statistical heterogeneity.

#### 2.5.7 Assessment of reporting biases

We will make visual asymmetry on a funnel plot via Egger methods if all included trials are more than 10 in the review, which is used to detect the latent reporting biases. The reviewers will use STATA 15 software to run the quantitative analysis of the Egger test if the image is unclear.

#### 2.5.8 Data synthesis

RevMan 5.3 will be applied to conduct a meta-analysis when there is a reliable and sufficient evidence. The rate ratio (RR) will be conducted to indicate extracted data for dichotomous outcomes while the mean difference (MD) for measurement data. The size will be expressed with a 95% confidence interval (CI) for analysis. If the significant statistical heterogeneity is not found, the fixed effects model will be used to computed RR and MD. If it is on the contrary, the random-effects model will be applied to present the data synthesis. When heterogeneity is found, the researchers will conduct the subgroup analysis or meta-regression analysis; otherwise, if meta-analysis cannot be performed, we will only make an analysis which includes the summary and the explanation of the characteristics and results of included studies.

### 2.6 Subgroup analysis and investigation of heterogeneity

The subgroup analysis will be conducted for the aim of exploring the resources of the heterogeneity, the following factors will be taken into account: inconsistent patients characteristic, classification of BA, disease course, different types of interventions, outcome measures, and other factor which cannot be predicted.

### 2.7 Sensitivity analysis

If necessary, sensitivity analysis will be applied to investigate funnel plot asymmetry. Sensitivity analysis will be conducted according to methodological qualities, sample size and the effect of missing data. The meta-analysis will be repeated after excluding high-risk bias studies and the outcomes will be compared with the original one.

### 2.8 Assessment of quality of evidence

The Grading of recommendations Assessment Development and Evaluation (GRADE) will be applied to evaluate the quality of the review.

## 3 Discussion

Hepatic fibrosis after Kasai portoenterostomy directly affects the survival rate of children with BA. Chinese medicine has certain potential and advantages in preventing and treating hepatic fibrosis. According to traditional Chinese medicine, the characteristics of BA are “dampness, stagnation, phlegm, deficiency”.^[15]^ YCSND is used to warm the interior, reinforce the yang, resolve dampness and reduce jaundice. The chief herb, Artemisiae Scopariae Herba (yin chen), can be used in treating all types of jaundice. The other chief herb is Aconiti Lateralis Radix Praeparata (fu zi). With its hot property and acrid flavor, fu zi has a very strong effect in warming and stimulating the Kidney yang, which can disseminates throughout the body to dispel coldness. The deputy, Zingiberis Rhizoma (gan jiang), warms the middle and dispels coldness, which stiffen the Spleen and stomach's functions of digestion and absorption. Tradition says that fu zi and gan jiang used in combination will have more efficient effect in warming the Kidney yang, which travels through the 12 regular meridians to warm the body. The assistant herb, Glycyrrhizae Radix Et Rhizoma (gan cao), invigorates Qi, strengthens the Spleen, reduces the side effect, and moderates the drying properties of the fu zi and gan jiang. These 4 herbs cooperate to play the role of warming the middle burner, resolving dampness, and reducing jaundice. YCSND is a good prescription for treating yin-type or damp-cold jaundice with Kidney yang deficiency. In the clinical treatment of BA, YCSND can improve the quality of life.^[16]^

However, a systematic review of YCSND in treating BA has not yet been published. This systematic review will be the first to provide a summary of the current state of evidence regarding the efficacy and safety of YCSND in treating children after Kasai portoenterostomy for BA. This evaluation will be useful for practitioners and patients with BA.

## Author contributions

Authorship: HX is the guarantor of the article. GC and HX conceived the idea for this study and drafted the protocol. CH and JL developed the search strategy. CH, JL, and RH will independently screen the potential studies and extract data. JL, RH, ZC and JZ will performed risk of bias assessment and finish data synthesis. GC will supervise the project and revised the manuscript. All review authors approved the final manuscript.

**Conceptualization:** Guoming Chen.

**Data curation:** Chuyao Huang, Jiaxin Lu, Ruilan Huang.

**Formal analysis:** Ziyin Chen.

**Methodology:** Jie Zhang.

**Project administration:** Guoming Chen.

**Resources:** Guoming Chen, Hua Xu.

**Software:** Ruilan Huang.

**Supervision:** Guoming Chen, Hua Xu.

**Writing – original draft:** Guoming Chen, Chuyao Huang, Jiaxin Lu, Ruilan Huang, Jie Zhang, Ziyin Chen.

**Writing – review & editing:** Guoming Chen, Hua Xu.

Hua Xu orcid: 0000-0003-2212-7347.
